# 同位素内标-气相色谱-高分辨双聚焦磁质谱法检测血清中多溴联苯醚

**DOI:** 10.3724/SP.J.1123.2021.10017

**Published:** 2022-04-08

**Authors:** Mengmeng WANG, Linna XIE, Ying ZHU, Yifu LU

**Affiliations:** 中国疾病预防控制中心环境与人群健康重点实验室, 中国疾病预防控制中心环境与健康相关产品安全所, 北京 100021; China CDC Key Laboratory of Environment and Population Health, National Institute of Environmental Health, Chinese Center for Disease Control and Prevention, Beijing 100021, China; 中国疾病预防控制中心环境与人群健康重点实验室, 中国疾病预防控制中心环境与健康相关产品安全所, 北京 100021; China CDC Key Laboratory of Environment and Population Health, National Institute of Environmental Health, Chinese Center for Disease Control and Prevention, Beijing 100021, China; 中国疾病预防控制中心环境与人群健康重点实验室, 中国疾病预防控制中心环境与健康相关产品安全所, 北京 100021; China CDC Key Laboratory of Environment and Population Health, National Institute of Environmental Health, Chinese Center for Disease Control and Prevention, Beijing 100021, China; 中国疾病预防控制中心环境与人群健康重点实验室, 中国疾病预防控制中心环境与健康相关产品安全所, 北京 100021; China CDC Key Laboratory of Environment and Population Health, National Institute of Environmental Health, Chinese Center for Disease Control and Prevention, Beijing 100021, China

**Keywords:** 气相色谱-高分辨双聚焦磁质谱, 液液萃取, 生物监测, 多溴联苯醚, 血清, gas chromatography-high resolution dual-focus magnetic mass spectrometry (GC-HRMS), liquid-liquid extraction (LLE), biological monitoring, polybrominated diphenyl ethers, serum

## Abstract

建立了同位素内标-气相色谱-高分辨双聚焦磁质谱(GC-HRMS)同时测定人体血清中14种多溴联苯醚(PBDEs)的方法。血清样品解冻后,取0.5 mL与^13^C标记的内标物进行混合,加入甲醇沉淀样品中的蛋白质,比较了3种酸化条件下的去脂效果和回收率,结果显示硫酸去脂效果最好;使用液液萃取法提取样品中的目标物,比较了不同萃取溶剂对回收率的影响,结果显示以正己烷(6 mL)-甲基叔丁基醚(6 mL)作为萃取溶剂效果最好;提取液经固相萃取柱净化和洗脱,比较了不同固相萃取柱和洗脱溶剂的净化效果和回收率,结果显示采用硅胶柱净化和用正己烷-二氯甲烷(1:1, v/v)洗脱时效果最好;洗脱液经氮吹近干后用正己烷复溶,经GC-HRMS测定。目标物经Rtx-1614色谱柱(30 m×0.25 mm×0.1 μm)分离,电压选择离子检测(VSIR)模式测定。BDE-209在0.40~25 μg/L、其他13种多溴联苯醚在0.08~5 μg/L范围内线性关系良好,相关系数>0.995,方法检出限为0.01~0.51 μg/L,定量限为0.04~1.70 μg/L,加标回收率为75.5%~120.7%,日内精密度为3.8%~10.9%(*n*=6),日间精密度为4.2%~12.4%(*n*=6)。应用该方法对采集的某地区15份青少年血清样本进行检测,结果显示14种PBDEs中,BDE-47检出率为100%,其他组分均未检出,说明该人群存在一定的PBDEs暴露。与现有文献报道方法相比,本方法样本需求量少,灵敏度、准确度较高,可对人血清中包括BDE-209在内的14种PBDEs同时测定,有效提高检测效率。本方法的建立可为我国开展多溴联苯醚对人群健康的影响提供技术支撑。

## Abstract (en)

A method for the determination of 14 polybrominated diphenyl ethers (PBDEs) in human serum using isotope internal standard-gas chromatography-high resolution dual-focus magnetic mass spectrometry (GC-HRMS) was developed. After thawed to room temperature, 0.5 mL serum samples were mixed with ^13^C-labeled isotopic internal standard. Subsequently, methanol was added to precipitate the proteins in the samples. The effects of three kinds of acids on the removal of cellulite from the serum samples and the corresponding recoveries were compared, and the results revealed that sulfuric acid was the most optimal. The target compounds were extracted by liquid-liquid extraction (LLE), and the effects of different extraction solvents on recoveries were compared. The results indicated that *n*-hexane (6 mL)-methyl *tert*-butyl ether (6 mL) was the best extraction solvent. The extracts were cleaned and eluted using solid phase extraction cartridges. Furthermore, the factors that influenced the cleanup effects and recoveries, including the solid phase extraction columns and elution solvents, were investigated in detail. The results indicated that the optimal conditions were cleanup with a silica gel column and elution with hexane-dichloromethane (1:1, v/v). The eluate was re-dissolved in hexane after being blown to near dryness using nitrogen. The detection of PBDEs was performed using GC-HRMS. The instrument conditions were optimized, and the capillary column used was an Rtx-1614 column (30 m×0.25 mm×0.1 μm). Helium was used as the carrier gas at a flow rate of 1.0 mL/min. The injector temperature was 290 ℃, and the oven temperature was programmed as follows: 150 ℃ for 2 min, 150 ℃ to 250 ℃ at 15 ℃/min, held for 1 min, 250 ℃ to 290 ℃ at 25 ℃/min, held for 3 min, and 290 ℃ to 320 ℃ at 25 ℃/min, held for 12.5 min. The injection volume was 1 μL in splitless mode. The samples were ionized in the positive electron ionization (EI) mode at 35 eV. Precursor ions and the production of each compound were identified using a voltage-selective ion detection (VSIR) program with a resolution of 10000. The ionization temperature was set at 280 ℃, and the transmission line temperature was set at 320 ℃. To ensure the integrity of the separation of low-brominated components, the column separation time was shortened, the response of high-boiling components was improved (BDE-190 and BDE-209), the decomposition of BDE-209 on the chromatographic column was effectively prevented, and the requirement of the simultaneous determination of multiple PBDEs was met. The method demonstrated good linearity in the range of 0.40 to 25 μg/L for BDE-209, and 0.08 to 5 μg/L for the other 13 PBDEs, with correlation coefficients greater than 0.995. The method detection limits (MDLs) were in the range of 0.01 to 0.51 μg/L, and the limits of quantification (LOQs) ranged from 0.04 to 1.70 μg/L. The recoveries of the 14 compounds ranged from 75.5% to 120.7%. The intra-day relative standard deviations (RSDs) were within 3.8%-10.9% (*n*=6) and the inter-day RSDs were within 4.2% to 12.4% (*n*=6). This method was successfully applied to the determination of 14 PBDEs in 15 serum samples from an adolescent population in an area. Notably, 1.86 to 4.66 ng/g lipid BDE-47 was detected in the serum samples with a detection frequency of 100%, and the other compounds were not detected. The results imply that the adolescent population in this region was exposed to some PBDE. Compared with the existing methods reported, this method has less sample demand and higher sensitivity and accuracy, can simultaneously determine 14 PBDEs, including BDE-209 in human serum, and effectively improve the efficiency of detection. This study offers a new method for studying the impact of polybrominated diphenyl ethers on population health in China.

多溴联苯醚(polybrominated diphenyl ethers, PBDEs),是一种持久性有机污染物(persistent organic pollutants, POPs),根据苯环上溴原子的取代个数和位置的不同,共有10类209种同系物。由于其阻燃性能良好,被广泛应用于纺织品、玩具、建筑材料和电子设备等产品中^[1,2]^。PBDEs的化学结构稳定,亲脂性强,容易释放到环境中,并通过食物链对生物体产生生物蓄积与生物放大作用,产生甲状腺毒性、神经毒性、内分泌毒性、生殖毒性、肝脏毒性、细胞毒性、致癌性等^[3,4,5,6,7,8,9,10]^。

PBDEs对人体健康的影响已成为世界范围内高度关注的问题,目前针对多溴联苯醚人群暴露情况的研究,分析样本主要为血液、母乳和各种组织(脂肪、胎盘等)^[11]^。由于多溴联苯醚是脂溶性化合物,在尿液中含量较低且多以羟基化代谢物的形式存在,脂肪组织的采样具有侵害性,且母乳和胎盘的采样仅限于一部分特殊人群,而血液样本相对较易获得,所以血液样本的测定是研究多溴联苯醚对人群健康影响的主要途径。

人体血清基质复杂,PBDEs含量较低,因此需提高富集效率并尽可能降低基质干扰,提高检测灵敏度。目前,液液萃取法、固相萃取法和加速溶剂萃取法是样品提取时较常使用的方法,样品净化主要使用凝胶色谱法和固相萃取柱净化法,检测方法主要有液相色谱-质谱法(LC-MS)、气相色谱-串联质谱法(GC-MS/MS)^[12,13]^、气相色谱-负化学源质谱法(GC-NCI/MS)^[14]^和气相色谱-高分辨双聚焦磁质谱法(GC-HRMS)^[15,16]^。LC-MS前处理步骤相对简便,但对PBDEs分辨能力较弱、灵敏度较低,更适合易热降解的高溴代多溴联苯醚的测定;GC-MS/MS、GC-NCI/MS选择性、灵敏度较高,对复杂基质抗干扰能力强,适用于痕量PBDEs的测定,但样本需求量较大,需采集2~5 mL血清样本;GC-HRMS同时备有静电场离子分析器和磁场质量分析器,因而使仪器同时具有能量聚焦和方向聚焦的双聚焦功能,灵敏度高、检出限低,适用于小体积样本中痕量和超痕量PBDEs的测定。目前常用的GC-HRMS样品前处理步骤中主要采用凝胶色谱和酸性硅胶柱对样品进行净化,其中凝胶色谱法样本需求量较大(2 mL),酸性硅胶柱对实验人员填装操作要求较高,且无法同时测定多种PBDEs组分(如BDE-209等),批量样品检测时效率较低。本方法探索使用少量血清(0.5 mL),采用GC-HRMS结合液液萃取和硅胶柱净化的方法,建立了人血清中14种PBDEs的测定方法,并用该方法对某地区15份青少年人群血样进行了检测,以期了解该地区青少年人群PBDEs的暴露水平。

## 1 实验部分

### 1.1 仪器、试剂与材料

Autospec Premier气相色谱-高分辨双聚焦磁质谱仪(美国Waters公司), JA2003电子天平(上海舜宇恒平公司), FV64氮吹仪(广州得泰公司), 600C离心机(北京白洋医疗器械有限公司), HyperSep Silica固相萃取柱(6 mL, 500 mg)(美国Thermo公司)。

正己烷、甲基叔丁基醚、二氯甲烷、甲醇、壬烷(色谱纯,美国Merck公司),超纯水(德国Merck公司),硫酸(分析纯,中国国药公司),标准参考物质SRM1958(美国NIST标准物质)。

14种PBDEs(包括BDE-17、BDE-28、BDE-47、BDE-66、BDE-71、BDE-85、BDE-99、BDE-100、BDE-138、BDE-153、BDE-154、BDE-183、BDE-190、BDE-209)购自美国AccuStandard公司;^13^C标记的10种PBDEs (包括^13^C_12_-BDE-28、^13^C_12_-BDE-47、^13^C_12_-BDE-77、^13^C_12_-BDE-99、^13^C_12_-BDE-100、^13^C_12_-BDE-138、^13^C_12_-BDE-153、^13^C_12_-BDE-154、^13^C_12_-BDE-183和^13^C_12_-BDE-209)购自美国Cambridge Isotope Laboratories公司。

血清样本:采集对象为某地区15名健康青少年,均告知研究目的并签署了知情同意书。

### 1.2 标准溶液配制

PBDEs标准使用溶液:以壬烷为溶剂配成BDE-209质量浓度为5 mg/L、其他13种目标物质量浓度为1 mg/L的标准使用液。

PBDEs内标使用溶液:以甲苯为溶剂配成^13^C_12_-BDE-209质量浓度为500 μg/L、其他9种^13^C标记的PBDEs质量浓度为50 μg/L的内标使用液。

### 1.3 实验方法

1.3.1 样品前处理

血清样品解冻后移取0.5 mL于12 mL玻璃离心管中,分别加入200 μL硫酸、0.5 mL甲醇和20 μL内标使用溶液后混匀。先加入6 mL正己烷充分摇振后,以3500 r/min离心10 min,收集上层有机相;再加入6 mL甲基叔丁基醚,重复萃取,合并两次萃取液,于40 ℃、5 Pa氮吹25 min至0.5 mL。依次用2 mL甲醇和2 mL正己烷活化硅胶固相萃取柱,将浓缩液转移到硅胶柱上,先收集流出液,再用10 mL二氯甲烷-正己烷(1:1, v/v)溶液洗脱,合并流出液与洗脱液,40 ℃氮吹30 min至近干。向试管中加入10 μL正己烷复溶,振荡混匀,转移至棕色进样小瓶中,待测。

1.3.2 色谱条件

色谱柱:Rtx-1614毛细管柱(30 m×0.25 mm×0.1 μm);进样方式:不分流进样;进样口温度:290 ℃;传输线温度:320 ℃;升温程序:初始温度150 ℃,保持2 min,以15 ℃/min升温至250 ℃,保持1 min,再以25 ℃/min升温至290 ℃,保持3 min,然后以25 ℃/min升温至320 ℃,保持12.5 min;载气:氦气,恒定流量1.0 mL/min;进样量为1 μL。

1.3.3 质谱条件

电子轰击(EI)离子源,源温:280 ℃;电子能量:35 eV;电压选择离子检测(VSIR);分辨率:10000。14种PBDEs及其同位素内标的质谱参数见表1。

**表1:** 14种PBDEs及其同位素内标的保留时间和质谱参数

Compound	Abbreviation	Retention time/min	Monitoring ion [M^+^] (m/z)	Internal standard
2,2',4-Tribromodiphenyl ether	BDE-17	7.82	405.8027/407.8002	^13^C_12_-BDE-28
2,4,4'-Tribromodiphenyl ether	BDE-28	8.02	405.8027/407.8002	^13^C_12_-BDE-28
2,2',4,4'-Tetrabromodiphenyl ether	BDE-47	9.51	483.7132/485.7111	^13^C_12_-BDE-47
2,3',4,4'-Tetrabromodiphenyl ether	BDE-66	9.75	483.7132/485.7111	^13^C_12_-BDE-77
2,3',4',6-Tetrabromodiphenyl ether	BDE-71	9.31	483.7132/485.7111	^13^C_12_-BDE-47
2,2',3,4,4'-Pentabromodiphenyl ether	BDE-85	11.52	563.6216/565.6196	^13^C_12_-BDE-100
2,2',4,4',5-Pentabromodiphenyl ether	BDE-99	11.00	563.6216/565.6196	^13^C_12_-BDE-99
2,2',4,4',6-Pentabromodiphenyl ether	BDE-100	10.71	563.6216/565.6196	^13^C_12_-BDE-100
2,2',3,4,4',5'-Hexabromodiphenyl ether	BDE-138	12.86	641.5322/643.5302	^13^C_12_-BDE-138
2,2',4,4',5,5'-Hexabromodiphenyl ether	BDE-153	12.20	641.5322/643.5302	^13^C_12_-BDE-153
2,2',4,4',5,6'-Hexabromodiphenyl ether	BDE-154	11.78	641.5322/643.5302	^13^C_12_-BDE-154
2,2',3,4,4',5',6-Heptabromodiphenyl ether	BDE-183	13.78	721.4406/723.4386	^13^C_12_-BDE-183
2,3,3',4,4',5,6-Heptabromodiphenyl ether	BDE-190	14.95	721.4406/723.4386	^13^C_12_-BDE-183
Decabromodiphenyl ether	BDE-209	22.42	797.3355/799.3329	^13^C_12_-BDE-209
2,4,4'-Tribromodiphenyl ether-^13^C_12_	^13^C_12_-BDE-28	8.00	417.8429/419.8409	
2,2',4,4'-Tetrabromodiphenyl ether-^13^C_12_	^13^C_12_-BDE-47	9.50	497.7514/499.7493	
3,3',4,4'-Tetrabromodiphenyl ether-^13^C_12_	^13^C_12_-BDE-77	10.11	497.7514/499.7493	
2,2',4,4',5-Pentabromodiphenyl ether-^13^C_12_	^13^C_12_-BDE-99	11.00	575.6619/577.6598	
2,2',4,4',6-Pentabromodiphenyl ether-^13^C_12_	^13^C_12_-BDE-100	10.70	575.6619/577.6598	
2,2',3,4,4',5'-Hexabromodiphenyl ether-^13^C_12_	^13^C_12_-BDE-138	12.85	655.5704/657.5683	
2,2',4,4',5,5'-Hexabromodiphenyl ether-^13^C_12_	^13^C_12_-BDE-153	12.19	655.5704/657.5683	
2,2',4,4',5,6'-Hexabromodiphenyl ether-^13^C_12_	^13^C_12_-BDE-154	11.77	655.5704/657.5683	
2,2',3,4,4',5',6-Heptabromodiphenyl ether-^13^C_12_	^13^C_12_-BDE-183	13.77	733.4809/735.4788	
Decabromodiphenyl ether-^13^C_12_	^13^C_12_-BDE-209	22.41	809.3757/811.3731	

### 1.4 质量控制

样品前处理环境应在每次实验开始前和结束后进行清理,避免有目标物残留。实验过程中所用玻璃离心管、试剂、进样小瓶、固相萃取柱、枪头均做空白对照实验,未检出14种待测PBDEs。

## 2 结果与讨论

### 2.1 分析条件优化

2.1.1 色谱柱的选择

目前PBDEs的气相色谱-质谱检测通常需用两根不同的非极性色谱柱完成。这是因为BDE-209热稳定性较差,易在色谱柱中分解,一般采用15 m或更短的色谱柱有利于缩短柱分离时间并提高响应,其他组分使用30 m的色谱柱进行分离,检测过程中需频繁切换色谱柱,效率较低。本研究采用Rtx-1614毛细管柱(30 m×0.25 mm×0.1 μm),相比常用的DB-5MS等非极性色谱柱,在保证低溴代组分分离完好的前提下,缩短柱分离时间,可有效防止BDE-209在色谱柱上的分解,可满足同时测定多种PBDEs的需求,提高检测效率。

2.1.2 色谱条件的选择

多溴联苯醚沸点较高,沸程较宽(310~425 ℃),程序升温过快,低溴代组分保留时间接近,不能较好分离;柱温过低,高沸点组分不能完全气化,响应会明显降低;柱温过高会加速柱流失,降低柱效,缩短色谱柱使用寿命,因此升温程序的最高温度不宜超过320 ℃。分别比较了程序升温最高温度为300、310和320 ℃时目标物响应情况,发现320 ℃时可明显提高高沸点组分(BDE-190和BDE-209)的响应(见图1),同时缩短了BDE-209的柱分离时间,防止其在色谱柱上的分解。

**图1:**
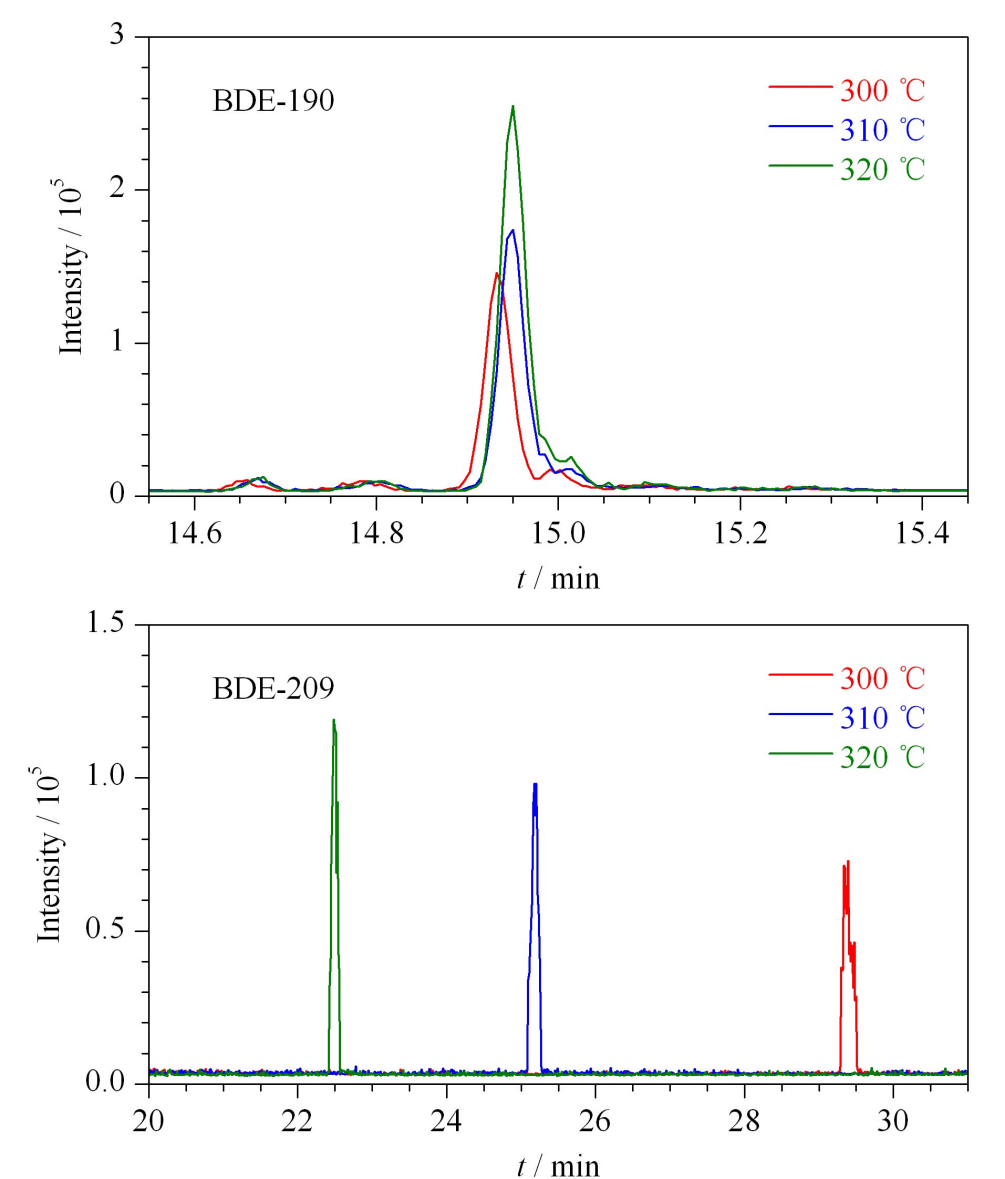
升温程序最高温度对部分目标化合物响应值的影响

经过优化试验,综合考虑各组分的分离度、灵敏度及分析时间等因素,最终选择初始温度150 ℃,保持2 min,以15 ℃/min升到250 ℃,保持1 min,再以25 ℃/min升到290 ℃,保持3 min,最后以25 ℃/min升到320 ℃,保持12.5 min的三阶升温模式。各组分分离度良好,14种PBDEs和10种相应同位素内标的色谱图见图2。

**图2:**
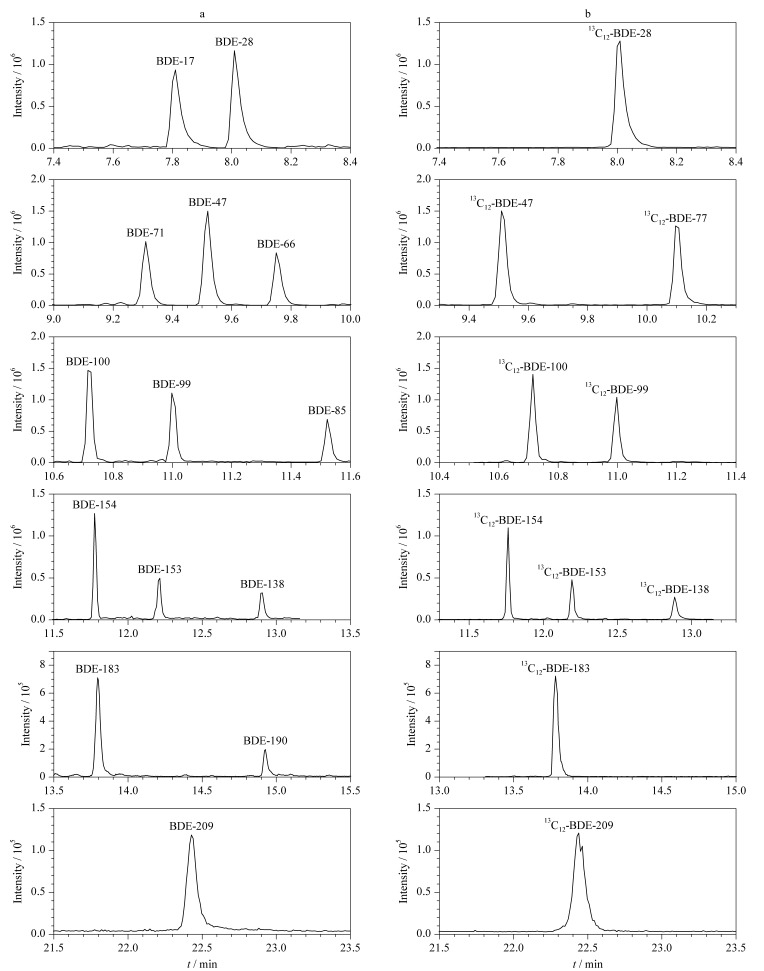
(a)14种PBDEs和(b)同位素内标的色谱图

2.1.3 质谱条件的选择

分别考察了离子源温度分别为280、290和300 ℃时,目标化合物的响应强度变化。温度较高时,相对分子质量较小的大部分化合物碎片离子较多,用于定性和定量的离子丰度下降;但相对分子质量较大的化合物离子丰度较高。综合这两方面的因素,本方法离子源温度设为280 ℃。

对传输线温度进行优化时发现温度变化对低溴代组分影响较小,对高溴代组分影响较大,尤其是BDE-209,随着传输线温度的升高不仅出峰时间加快,响应也明显提高(见图3),最终传输线温度设为320 ℃。

**图3:**
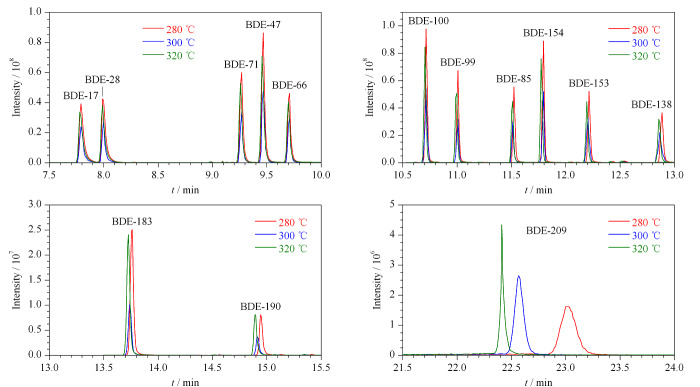
传输线温度对目标化合物响应值的影响

在特征碎片离子的确定过程中,重点优化了BDE-209特征碎片离子的选择。通过对比不同碎片离子响应,最终选择响应最强的BDE-209脱掉两个溴离子的碎片离子[M-2Br]^+^,即*m/z* 799作为监测离子(见图4)。

**图4:**
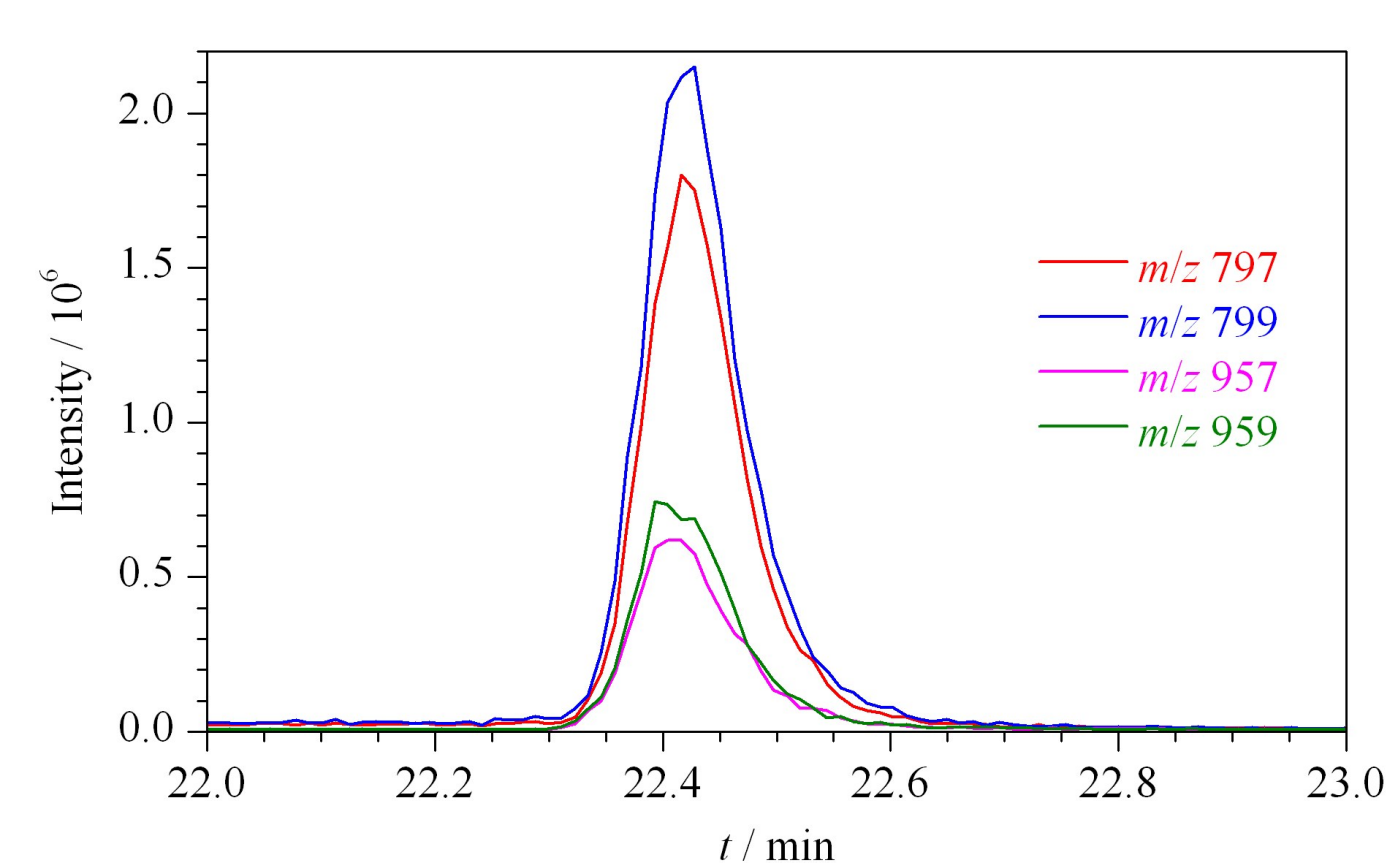
监测离子对BDE-209响应值的影响

### 2.2 前处理条件优化

2.2.1 萃取条件的选择

酸化条件 由于人体血液样品中多溴联苯醚含量低,且样品基质复杂,常采用多层硅胶柱进行净化^[17]^。但由于无相应的商品化产品,需人工填装,过程繁琐,重复性较差,大批量样本检测时效率较低。本实验采用柱前加酸的方式完成去脂净化,分别比较了甲酸、硫酸和盐酸的去脂效果和回收率。结果表明,甲酸的效果较差,固相萃取净化时易堵塞萃取柱,硫酸和盐酸去脂效果和回收率均较好,但采用硫酸酸化后,高溴代组分如BDE-190和BDE-209的响应更好,故选用硫酸酸化法去脂。萃取溶剂 选择常用的萃取溶剂正己烷和甲基叔丁基醚,比较了正己烷(12 mL)、甲基叔丁基醚(12 mL)和正己烷(6 mL)-甲基叔丁基醚(6 mL)对回收率的影响。结果(见图5)表明,单独使用正己烷或甲基叔丁基醚时,BDE-17等低溴代组分的回收率较差。最终选用正己烷(6 mL)-甲基叔丁基醚(6 mL)作为萃取溶剂。

**图5:**
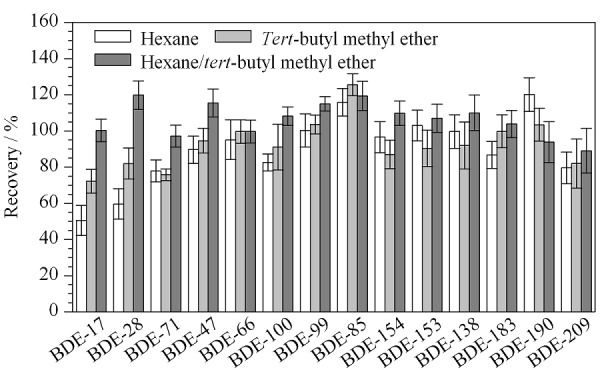
萃取溶剂对目标化合物回收率的影响(*n*=6)

2.2.2 固相萃取柱的选择

液液萃取后需使用固相萃取柱对提取液进行富集净化,不同类型的填充料对实验结果影响较大。本研究比较了填充料为硅胶和氧化铝的固相萃取柱的净化效果和对回收率的影响。结果表明,相比于氧化铝固相萃取柱,使用硅胶固相萃取柱时各组分回收率更好(见图6)。

**图6:**
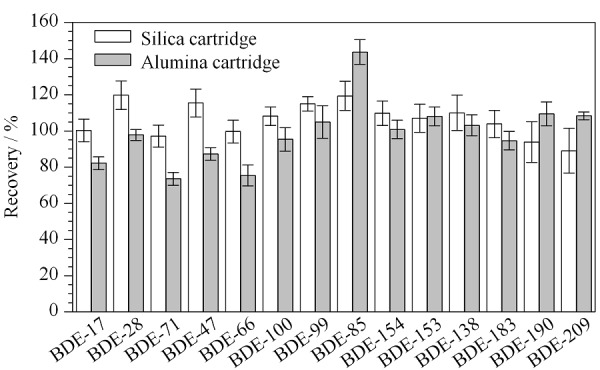
固相萃取柱对目标化合物回收率的影响(*n*=6)

2.2.3 洗脱溶剂的选择

洗脱溶剂的极性影响目标物的洗脱效果。本方法比较了常用洗脱溶剂正己烷、二氯甲烷和正己烷-二氯甲烷(1:1, v/v)对回收率的影响。结果表明,洗脱溶剂选择正己烷-二氯甲烷(1:1, v/v)时效果最好(见图7)。

**图7:**
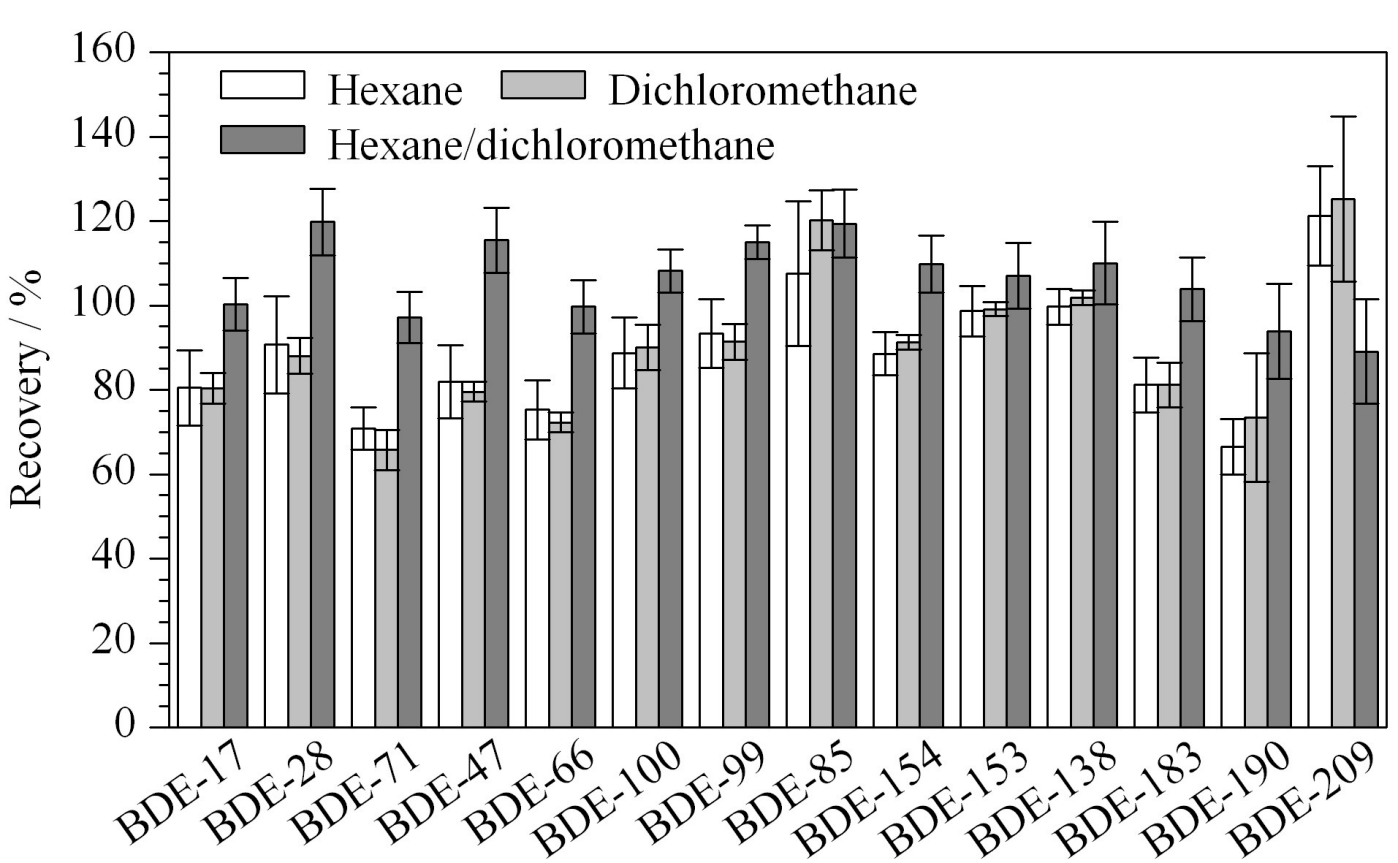
洗脱溶剂对目标化合物回收率的影响(*n*=6)

### 2.3 方法学考察

2.3.1 线性范围和检出限

用正己烷配制目标分析物质量浓度分别为4、10、25、50、100和250 μg/L,内标质量浓度为50 μg/L的标准系列溶液(BDE-209及其对应内标的质量浓度为上述质量浓度的5倍),换算到人血清中的质量浓度分别为0.08、0.2、0.5、1、2和5 μg/L(BDE-209质量浓度为上述质量浓度的5倍)。按照本方法仪器分析条件进行测定,以目标物与相应内标离子峰的峰面积之比为纵坐标,目标物与相应内标质量浓度之比为横坐标,绘制校准曲线。结果显示,14种PBDEs的线性相关系数均大于0.995。参照美国疾病预防控制中心关于方法检出限计算的要求,对质量浓度为预估方法检出限3~5倍的样品重复测定*n*次(*n*≥7),以3倍标准偏差为方法检出限,10倍标准偏差为定量限。本方法检出限为0.01~0.51 μg/L,定量限为0.04~1.70 μg/L,结果见表2。

**表2:** 14种PBDEs的线性范围、回归方程、相关系数、方法检出限(MDL)和定量限

Compound	Linear range/(μg/L)	Linear equation	r	MDL/(μg/L)	LOQ/(μg/L)
BDE-17	0.08-5	y=0.7414x+0.0435	0.999	0.02	0.07
BDE-28	0.08-5	y=0.7717x+0.0006	0.999	0.02	0.06
BDE-47	0.08-5	y=1.0190x+0.009	0.999	0.01	0.04
BDE-66	0.08-5	y=0.6454x-0.0144	0.999	0.03	0.09
BDE-71	0.08-5	y=0.7503x+0.0230	0.999	0.02	0.08
BDE-85	0.08-5	y=0.5084x-0.0226	0.998	0.03	0.09
BDE-99	0.08-5	y=0.8007x+0.0084	0.999	0.03	0.10
BDE-100	0.08-5	y=0.9413x-0.0028	0.999	0.02	0.06
BDE-138	0.08-5	y=1.0211x-0.0127	0.999	0.04	0.13
BDE-153	0.08-5	y=0.9191x+0.0155	0.999	0.03	0.10
BDE-154	0.08-5	y=0.9278x+0.0002	0.999	0.03	0.09
BDE-183	0.08-5	y=0.8414x-0.0075	0.999	0.04	0.12
BDE-190	0.08-5	y=0.2424x-0.0073	0.999	0.08	0.27
BDE-209	0.40-25	y=0.5393x+0.0363	0.999	0.51	1.70

y: peak area ratio of quantitative ion of the analyte to IS; x: mass concentration ratio of the analyte to IS.

2.3.2 加标回收率和精密度

以胎牛血清为基质进行低、中、高3个水平的加标回收试验,加标质量浓度分别为0.2、0.4、0.8 μg/L(BDE-209质量浓度为上述质量浓度的5倍),每个水平平行测定6次,考察日内精密度,连续测定6天,考察日间精密度。14种PBDEs的平均回收率为75.5%~120.7%,日内和日间精密度分别为3.8%~10.9%和4.2%~12.4%,结果见表3。

**表3:** 14种PBDEs在血清中的加标回收率和日内、日间精密度(n=6)

Compound	0.2 (1.0) μg/L^*^		0.4 (2.0) μg/L^*^		0.8 (4.0) μg/L^*^		Intra-day RSD/%	Inter-day RSD/%
	Recovery/%	RSD/%	Recovery/%	RSD/%	Recovery/%	RSD/%		
BDE-17	92.5	4.0	82.9	4.0	100.2	6.2	4.8	8.4
BDE-28	118.6	2.5	119.3	4.1	120.7	6.6	4.4	8.4
BDE-47	105.2	2.8	113.2	2.5	97.2	6.3	3.8	4.8
BDE-66	118.9	3.8	116.5	1.9	115.4	6.6	4.1	8.0
BDE-71	75.5	5.6	81.6	4.1	99.7	6.4	5.3	12.4
BDE-85	119.7	3.7	116.8	3.7	108.2	4.7	4.0	9.5
BDE-99	102.3	11.9	109.3	2.2	115.0	3.4	5.8	10.9
BDE-100	100.1	7.5	106.6	1.4	119.4	6.7	5.2	5.7
BDE-138	98.7	6.4	99.9	4.6	109.8	6.1	5.7	9.1
BDE-153	88.8	5.7	98.7	2.6	107.1	7.3	5.2	8.6
BDE-154	94.7	11.4	104.3	2.2	110.0	8.9	7.5	5.2
BDE-183	88.8	10.4	97.8	3.1	103.8	7.3	6.9	9.6
BDE-190	88.1	15.4	97.1	5.5	93.9	11.9	10.9	7.6
BDE-209	89.1	13.9	89.8	5.2	97.3	3.8	7.6	4.2

* The data in brackets are mass concentrations of BDE-209.

2.3.3 正确度评价

用本方法测定美国NIST人血清标准参考物质SRM1958,与其参考值进行比较,结果均在参考值范围内(见表4)。

**表4:** SRM1958测定值与参考值比较(*n*=6)

Compound	Experimental result/ (ng/g)	Certified value/ (ng/g)
BDE-17	0.453	0.458±0.032
BDE-28	0.466	0.462±0.019
BDE-47	0.656	0.651±0.029
BDE-66	0.471	0.440±0.041
BDE-99	0.484	0.492±0.015
BDE-100	0.489	0.475±0.027
BDE-153	0.412	0.455±0.054
BDE-154	0.418	0.441±0.039
BDE-183	0.425	0.453±0.042

NIST: National Institute of Standards and Technology.

### 2.4 方法比较

对本方法和文献报道的方法进行比较,本方法共有3个优点:(1)样本需求量少:由于血清采集较难获得大量样本,本方法仅需0.5 mL血清样本,更适用于PBDEs暴露对人体健康影响的研究。(2)检测效率显著提高:目前PBDEs的检测需用两根不同的非极性色谱柱完成,实际应用过程中需要频繁切换色谱柱,本方法可对包括BDE-209在内的14种PBDEs同时测定,可有效提高检测效率。(3)方法灵敏度高:与文献报道方法^[18,19,20]^相比,本方法在减少取样量的基础上,可同时测定高溴代组分(如BDE-209),方法检出限较低,可满足实际样品检测的需求。

比较结果见表5。

**表5:** 本方法与文献报道的血清中PBDEs检测方法的比较

Method	Sample volume/ mL	MDLs/(μg/L)													
		BDE- 17	BDE- 28	BDE- 71	BDE- 47	BDE- 66	BDE- 100	BDE- 99	BDE- 85	BDE- 154	BDE- 153	BDE- 138	BDE- 183	BDE- 190	BDE- 209
GC-MS/MS^[18]^	1	0.03	0.03	/	0.06	0.03	0.04	0.05	/	0.03	0.04	/	0.05	0.2	/
GC/ITD-MS/MS^[19]^	1	0.4	0.3	/	0.2	0.07	0.5	0.1	0.3	0.2	0.1	/	1.3	/	/
GC-HRMS^[20]^	2~5	/	0.01	/	0.02	/	0.02	0.03	0.04	0.02	0.02	0.03	0.04	/	/
This method	0.5	0.02	0.02	0.02	0.01	0.03	0.02	0.03	0.03	0.03	0.03	0.04	0.04	0.08	0.51

/: not reported.

### 2.5 实际样品测定

对某地区15份青少年人群的血清样本进行检测,BDE-47检出率为100%,含量范围为1.86~4.66 ng/g(以脂重计),其他13种PBDEs均未检出。根据文献报道,中国普通人群血清中PBDEs单体含量在0.01~14.6 ng/g(以脂重计)之间^[21,22,23]^,其中检出率最高的为BDE-47。本研究样品测定的结果与现有文献结果基本一致。

## 3 结论

本文通过对色谱条件、质谱条件和固相萃取柱类型、萃取和洗脱溶剂组成等参数进行优化,建立了液液萃取-气相色谱-高分辨双聚焦磁质谱测定人体血清中14种PBDEs的分析方法。本方法样本量需求较少(0.5 mL),操作简便,灵敏度和精密度可满足实际样品大批量检测的需求。
